# The active participation of elderly patients in traditional Chinese medicine consultations as means to creating a community of practice: A case study in Hong Kong

**DOI:** 10.3389/fpsyg.2022.948988

**Published:** 2022-10-03

**Authors:** Jack Pun

**Affiliations:** Department of English, College of Liberal Arts and Social Sciences, City University of Hong Kong, Kowloon, Hong Kong SAR, China

**Keywords:** traditional Chinese medicine, patient-centred care, evidence-based practice, community of practice, doctor-patient interaction

## Abstract

Despite its historic role in Chinese society and its popularity with an elderly Chinese population, limited research has explored the discursive practices of Traditional Chinese Medicine (TCM). Our analysis of practitioner–patient interactions illustrated the reasons why TCM is widely welcomed by a specific patient group. This paper adopted the concept of Community of Practice (CoP) as a theoretical framework to document how patients interact with TCM practitioners to construct meaning through a process of participation and reification. This study aimed to explore how patients in a medical consultation context developed an understanding of their conditions and how they strategically used medical terminology to enact meaningful exchanges to realise their CoP. An inductive qualitative discourse analysis was conducted to for nine elderly patients (i.e., age > 65) in Hong Kong to demonstrate the role that a CoP played in a joint process of knowledge construction to promote a patient-centred practice and foster the active participation of the patients. TCM practitioners can develop specific communication skills to promote their patients’ CoP, which will improve patient-centred care, empower patients to actively engage in their treatments and result in higher levels of patient satisfaction.

## Introduction

An ageing population and the management of elder care present tremendous challenges for many stakeholders in Hong Kong, as these challenges co-occur with a dramatic reduction in birth rates, a lower mortality rate and a higher life expectancy. By 2040, 20% of the population of Hong Kong will be over the age of 65, with the median age expected to rise to 46.1 years and the old-age dependency ratio reaching 42.5 [Census and Statistics Department (C&SD). Hong Kong Special Administrative Region, 2017]. However, there is no clear policy for the active management of ageing and elder care, particularly in terms of healthy ageing for the elderly and how best to monitor their health within their communities. As traditional Chinese medicine (TCM) services are widely available in the Hong Kong public healthcare system, the vast majority of elderly patients prefer and opt for TCM over Western medicine in a primary care context (Chung et al., 2009), which is congruent with the results of a recent survey of Hong Kong’s senior citizens [Census and Statistics Department (C&SD). Hong Kong Special Administrative Region, 2017; Wong et al., 2017]. Similarly, another major Hong Kong study found that TCM practitioners managed 64% of patient complaints and chronic illnesses, with 30% of those referring to those with continuing Western-based medical treatment (the most common types of which are musculoskeletal issues, hypertension and diabetes; Wong et al., 2017). Communication is essential to ensure patient satisfaction as the majority of patients do not possess medical knowledge. Patients can only evaluate how their medical practitioner communicates and place more emphasis on the level of communication they receive (Browne et al., 2010).

Accordingly, it is important to understand both patients’ interpersonal concerns and their physical discomfort. It is likely that many elderly patients in Hong Kong choose TCM treatment because they believe that TCM practitioners can provide better long-term clinical outcomes and modification to their lifestyle (Census and Statistics Department (C&SD). Hong Kong Special Administrative Region, 2017; Pun et al., 2019b). This paper adopted the theoretical framework of Community of Practice (CoP) to document how patients interacted with their TCM practitioners to construct meaning through participation and reification. This study explored how patients developed an understanding of their conditions and strategically used medical terminology to conduct meaningful exchanges and accomplish their CoP in a medical consultation context.

## Background

Traditional Chinese Medicine (TCM) is a growing worldwide phenomenon. Despite its historic role in Chinese society, there remains only limited research on the nature of medical interactions between the patients and the practitioners in the Chinese context, especially those take place in the contemporary period (Yip and Zhang, 2020). During TCM consultations, doctors examine their patients and provide diagnoses which identify the imbalances in the pulse, tin he life force or energy and in the organs (Pun et al., 2019b, p. 183). This type of comprehensive account of the multiple bodily senses and organs differs from patients’ experiences of Western Medicine (WM), as a TCM approach takes a holistic view of both the patient’s physical body and their environment. TCM practitioners emphasise the psychosocial aspects of their patients’ lives, instead of solely assessing the patient’s illness or symptoms (Chan, 1995; Zhou and Nunes, 2012). Moreover, TCM practitioners often provide interpersonal engagement and emotional support to their patients [Wang, 2010; Zhang and Sleeboom-Faulkner, 2011; Census and Statistics Department (C&SD). Hong Kong Special Administrative Region, 2017]. This is perhaps due to the emphasis that TCM practitioners place on patient-centred communication, in contrast to WM practices (Wang, 2010). The patients and the practitioners construct the consultation collaboratively and small talks between them transform their relationship to a more symmetrical one (Yip, 2020). In a study, it is shown that directness in the advice given by the TCM practitioners are also considered appropriate by the patients (Yip, 2020). Absent from WM practices worldwide, TCM patients and doctors usually have a close relationship as they may visit the same doctor continuously [Census and Statistics Department (C&SD). Hong Kong Special Administrative Region, 2017]. A possible reason for this phenomenon visiting the same doctor could offer a more well-communicated and personalised consultation (Pun and Wong, 2022). Developing a close doctor–patient relationship improves the patient’s clinical and psychosocial outcomes, facilitates a shared decision-making process, and leads to higher levels of patient satisfaction (Zhou and Nunes, 2012; Pun et al., 2019b; Pun, 2020).

TCM is well established in Hong Kong. In 1999, the Chinese Medicine Ordinance which contains the regulation of practice of local traditional Chinese medicine practioners (CMP) was passed in the Legislative Council (Chinese Medicine Council of Hong Kong, 2020).The same year also marked the establishment of Chinese Medicine Council of Hong Kong which was responsible for the implementation of the measures that regulate Chinese medicine in Hong Kong (Department of Health, 2022). Currently, there are 10,449 CMP in Hong Kong (Pun and Wong, 2022). Ng et al. report that there are also changes of the popularity of TCM in Hong Kong throughout 1997 to now (Ng et al., 2022). While it was popular when the colonial period of Hong Kong has just begun as people are doubtful about the WM it went through a period of decline around the Second World War thanks to its increasing time and the considerable of preparation time. Followed by the registration and regulation of TCM in Hong Kong that took place in these recent decades, WM and TCM are both considered popular and accessible in the city (Ng et al., 2022).

In Hong Kong, health care is a unique mix of Western and Chinese values, and as such, it offers a comparative basis to evaluate cross-cultural expectations, practices and experiences between the East and the West in terms of communication strategies of the clinician, patient expectations and their satisfaction. These interchanges also provide a unique opportunity to evaluate transfers between TCM and WM practices. Chung et al. revealed that elderly patients in particular were more satisfied with TCM practitioners than with WM doctors (Chung et al., 2009). TCM has thus been regarded as one of the most important initiatives for the promotion of primary care in the community. This was reflected in the HKSAR Government’s recent policy to address the further development of TCM, which would offer essential medical services in a primary care setting. The first TCM hospital is anticipated to open in 2025. This accelerated development arose from the high patient loads in public hospitals, the potential benefits of shifting patients to TCM during flu seasons, and the usage of TCM for pain management and to manage and treat chronic diseases such as diabetes mellitus and cancer.

Using a conversational analysis approach, Pun et al. conducted the first study on doctor–patient communication in TCM practice in Hong Kong to explore how the TCM practitioners interacted with their patients in TCM clinics (Pun, 2020; Pun and Wong, 2022). They found that TCM practitioners strategically used a range of linguistic strategies to understand how their patients understood their own conditions, to promote patient-centred care and to co-create a joint decision-making process for clinical treatment. Pun and Chor illustrated how TCM practitioners skilfully used a range of questions (i.e., general questions, biomedical questions, psychosocial questions) and formats (i.e., open-ended, closed-ended, clarification and tracking) to create a space for patients to discuss their illnesses and their primary concerns (Pun and Chor, 2020). Pun and Chor defined these moments as a form of ‘*touch*’ (this term is used to represent the nature of holistic TCM interactions) to co-construct an understanding of the patient’s condition (Pun and Chor, 2020). In these instances, the TCM practitioners interacted with their patients to explore their symptoms or ‘*Zheng*’, and opened a space for patients to participate in the ongoing discourse to clarify, discuss and understand their illnesses. Pun and Chor’s work is congruent with current studies on the different types of doctor–elderly patient communication in both TCM and WM practices, including psychological and lifestyle exchanges, non-medical small talk, discussions around emotions and other open questions (Jin and Tay, 2017). According to these studies, TCM practitioners developed positive therapeutic relationships with their patients by demonstrating their concern and providing reassurance to their patients. An ongoing discursive practice of this nature can result in a longer-term relationship between a TCM practitioner and their patients. The importance of effective doctor–patient communication has been widely discussed in the English-speaking world; however, there have been no studies on TCM practitioners’ strategic communication skills in the context of their practice, nor on the role that a patient’s CoP plays in their understanding of their conditions. The next section introduces CoP as a framework of analysis.

### Community of practice

CoP is a sociocultural approach that views learning as form of participation and a process that allows participants to interact with others (Pavlenko and Lantolf, 2000), as participation is believed to play an important role in facilitating the learning process (Van-Lier, 2000). According to Van Lier, the quantity of understandable information alone does not lead to learning; accordingly, ‘the opportunities for meaningful action that the situation affords’ should be emphasised (Van-Lier, 2000, p. 252). From this perspective, learning can be considered to be a form of participation, which suggests that researchers can observe participant interactions to analyse the learning process (Pun et al., 2019a). Lave and Wenger introduced the concept of CoP and argued that learning is a ‘legitimate peripheral participation in situated learning’ (Lave and Wenger, 1991, p. 29). From this perspective, the inclusion of individual and community participants transforms the nature of their participation, as they transition from being apprentices at the margins to working as competent members of their language communities (Hall, 1993). Thus, the establishment of a CoP consists of three main criteria: shared goals or interests between the members of a community; a shared community in which members can jointly learn and interact; and a practice wherein members develop a shared repertoire of resources such as their experiences, tools, styles, specific forms of language register, artefacts and operating procedures. As summarised by Li et al., three interrelation dimensions circumscribe CoP, which are ‘mutual engagement’, ‘shared repertoire’ and ‘joint enterprise’ (Li et al., 2009). The former two elements are particularly relevant in analysing patient and doctor relationship and interaction with CoP, which was the aim of this study. Another feature of the concept of CoP that are meaningful to this study was knowledge being shared among individuals in a CoP group (Li et al., 2009). In a TCM consultation, the patient interacts with a TCM practitioner in the interest of a shared goal, to explore the condition of one’s body. These medical interactions become the context in which patients develop an understanding of their conditions. Patients will gradually use more medical terminology develop a shared repertoire of resources. CoP plays a role in this joint process of knowledge construction and the promotion of a patient’s accumulated knowledge. This shared repertoire is necessary to facilitate purposeful activity and for the construction of new meanings, knowledge and practices over time. Within a CoP, learning occurs through a ‘negotiation of meaning’ where meaning is constructed through the process of participation and reification (Wenger, 1999). In this framework, participation refers to the socially constructed phenomenon of identity formation of becoming a member of a community. Reification refers to ‘the process of giving form to our experience by producing objects that congeal this experience into “thingness”’. More simply, it refers to the process of transforming one’s experience into a concept, object, tool or label (Wenger, 1999, p. 58).

This study explored how patients in the context of a medical consultation developed an understanding of their conditions and strategically used medical terminology in the context of TCM to conduct meaningful exchanges to enact and co-create their CoP. An inductive qualitative discourse analysis was conducted to examine the role that CoP played in the joint process of knowledge construction and active participation in the patient centred TCM journey of 10 elderly patients (i.e., age > 65) in Hong Kong. If TCM practitioners develop their communication skills to promote a CoP for their patients, they will attain higher levels of patient-centred care, empower their patients to become actively engaged in their treatments and achieve higher levels of patient satisfaction.

## Methodology

Ethnography and discourse analysis were combined with an inductive qualitative design to form the methodology of this study (Pun et al., 2017, 2019b).

### Participants

Currently, 10,499 Chinese medicine practitioners registered in Hong Kong from both university clinics and local licensed community. Both types of clinics shared similarities in patient profiles, staff mixes and treatment protocols that are governed by the Chinese Medicine Council of Hong Kong. One TCM registered Cantonese-speaking practitioner and the patients were recruited in a university-operated primary care clinic in Hong Kong. The TCM clinic adopts and practices the two fundamental theories of health management of Chinese medicine: ‘prevention before disease onset’ as the center’s principal aim and ‘preventing diseases when there are none; speeding up recovery when the disease is minor; and preventing chronic disease progression’ as its goals. The Inclusion criteria for TCM practitioner are: Cantonese-speaking, possess a valid Practicing License of Registered Chinese Medicine Practitioner and registered as Chinese Medicine Practitioner issued by the Chinese Medicine Council of Hong Kong; have at least 1 year of post-registration clinical experience in clinic setting. During the study period, patients of the participating TCM practitioner who meet the inclusion criteria (i.e., Chinese adults able to communicate in Cantonese) were recruited. These patients who aged 65 or above with chronic diseases were invited to participate in this study. All the participants participated on a voluntary basis.

## Data

Ten patient participants and one TCM practitioner were recruited for this study through convenient sampling technique. The TCM practitioner was also a university staff responsible for training TCM practitioners, with around 10 years working experience, and 9 patients’ consultation data were included in this study. The included TCM patients mainly presented with chronic diseases (the most common types of which are Musculo-skeletal, upper respiratory with hypertension and diabetes, allergic rhinitis, depressive disorders, cardiovascular disease, or asthma) and they have been received TCM treatments in the past 12 months.

We conducted non-participating observations and audio recorded the interaction between TCM practitioner and the patients inside the clinic. Researcher left the recorder inside the consultation room without being present during the consultation as to make the TCM practitioner and patient feel at ease. Prior to the consultation, participants received a verbal explanation of the research project’s aims and objectives and were informed of the confidentiality protocols and their right to withdraw from the study. Participants were also informed that their participation was voluntary and that withdrawing from the study would not affect their current or future relationship with the clinic. The confidential recordings were transcribed and fully anonymised. In total 11 participants agreed to participate in this study and provided their written consent. The research team undertook 12 h of direct observations of the TCM practitioner–patient interactions in the consultation room. The patient consultations for all 10 patient participants were recorded, transcribed, translated into English, and analysed.

### Analytical framework

To address the gaps in the research mentioned above, this study adapted Lave and Wenger’s model (Lave and Wenger, 1991) of CoP and conducted an in-depth qualitative discourse analysis to capture the specific moments of meaning-making between elderly patients and their TCM practitioner. This study particularly aimed to provide micro-level analyses of how patients and TCM practitioners co-constructed their understanding of the patient’s condition and maintain the patient’s CoP. Roos observed that such phenomena illustrated that CoP was essential to activities designed to learn about the patient’s condition, such as the diagnosis of prostate cancer, as patients and medical practitioners co-constructed medical meaning by drawing upon their expanding medical repertoires and other semiotic resources to comprehend and understand the patient’s medical condition (Pun et al., 2017). The co-construction of knowledge that Roos described is congruent with Lave and Wenger’s notion of ‘apprenticeship’ and to the concept of a CoP (Lave and Wenger, 1991; Roos, 2003). Patients can establish their understanding of their conditions by using medical language and modes recognised by the TCM community, which demonstrates the vocabulary of a TCM patient apprenticeship. Thus, the ability to communicate medical ideas and to shift between everyday language and medical language, translanguaging from one semiotic mode to another, is essential to patient-centred care (Epstein and Street, 2007).

### Data analysis

All of the data were transcribed, translated into English and analysed using the *NVivo* qualitative data analysis software package. Ethnographic discourse analysis was conducted to identify the structure and communication patterns of TCM practitioner–patient interaction (i.e., question types, turn-taking, pauses; Wenger, 1999; Pun et al., 2019b). To quantify the observed patterns, we used a coding system to capture the sequence of doctor–patient communications based on previous communication studies in Hong Kong (Van-Lier, 2000). Two bilingual research assistants cross-referenced the original audio recordings with the translations and transcription to perform a final review and ensure the accuracy of the medically related terms and expressions. The data was independently coded by two raters according to a coding sheet, and they established an inter-rater reliability of (k > 0.8) using Cohen’s Kappa coefficient.

Using the ethnographic discourse approach, we unpacked the quality of the interaction sequences between the TCM practitioners and their patients about how medical information is communicated and exchanged between the two parties. For examples, how the TCM practitioners initiate, respond to and follow-up with the patient’s answers. We also focus on the TCM practitioners’ Cantonese when they are giving for clinical explanations of diagnoses and treatments, and patients’ Cantonese expressions for their symptom descriptions. Such analysis includes an investigation of interpersonal aspects, such as the way the patients are able to ask questions, raise concerns and seek clarifications from their doctors. We can then trace the flow of communication using the concept of CoP in building patient’s understanding of their medical conditions at different stages of their journeys (e.g., by taking patient histories, making diagnoses, and translating medical information). We conducted several review rounds to compare, sort and recode as we looked for connections between the coded segments.

## Results

### Managing uncertainty during collaborative decision-making to achieve consensus

By confirming the initial details and prompting the patient to provide additional information (as seen in line 55), the patient participated in the communicative process could understand that the practitioner was trying to understand the issue and actively listening to the patient’s concerns with empathy (Oates et al., 2000; Stewart, 2001; Hong and Oh, 2020). Although the TCM practitioner may initially be uncertain of the patient’s level of pain and its duration, they could achieve mutual certainty by clarifying and verifying the patient’s explanations. As a part of the consultation, the TCM practitioner wanted to know more about the patient’s acute shoulder and neck pain, and particularly how long the pain had persisted. In Table 1, line 54 of the transcription, the practitioner verified their understanding of the answer by repeating it back to the patient. The patient then clarified her answer by illustrating how and when the pain is at its most intense, such as when performing the exercises referred to in line 55. Both parties thus achieved a degree of certainty regarding the patient’s level of shoulder and neck pain. The patient’s overall satisfaction would have been increased had the TCM practitioner lowered the patient’s level of uncertainty and anxiety (Wanzer et al., 2004; Dutta-Bergman, 2005; Trummer et al., 2006).

**Table 1:** Example of managing uncertainty during collaborative decision-making to achieve consensus.

Turn	Speaker	Text	Coding	Topic
52	D	=But you felt actually, was the pain a consecutive one or did it stop for a while?	Y/N Q (clarification)	Gripping pain (duration)
53	P	=Consecutive, consecutive.	Answer	Gripping pain (duration)
54	D	[Consecutive?	Y/N Q (checking)	Gripping pain (duration)
55	P	=Only when you turn to that area. It would feel painful. It was the most obvious when you do sit ups. When you get up after laying down, you’d use this area, and this was the most painful. =	Elicited answer	gripping pain when exercising

### Defining uncertainty in collaborative decision-making

In the consultation (Table 2), a TCM practitioner clarified the uncertainty of the patient, which could not only ensure patient’s compliance to the medical instruction but also expand the shared repertoire. Patient-centred communication encouraged the patient to follow the recommendations and medications, which led to a successful medical treatment and an improvement in the patient’s health (Burgoon et al., 1987; Wanzer et al., 2004; Bauer et al., 2014). During a conversation regarding the kinds of soups that the patient could prepare at home, in Table 2, in line 230 the TCM practitioner advised the patient not to include the herb Chinese Angelica in her soup. To clarify the health effects of the soup and the materials the practitioner suggested, in line 231, the patient initiated a yes/no question to better understand the health aims of the soup and to prompt the practitioner to elaborate on its benefits. Providing these suggestions demonstrated the practitioner’s active listening skills and imparted knowledge to the patient through their shared repertoire of TCM terminology, such as “clearing up heat” (清熱).

**Table 2:** Example of defining uncertainty in collaborative decision-making.

Turn	Speaker	Text	Coding	Topic
*228*	*D*	Thin pork. Also, Chinese Foxglove, Szechuan Lovage, solomonseal rhizome, lady bell root can be consumed by children as well.	Statement (advice)	TCM medication
*229*	*P*	Yes.		
*230*	*D*	But not Chinese Angelica. =	Statement (clarification)	TCM medication
*231*	*P*	=This soup aims at nursing the kidney and clearing up heatness?=	Y/N Q initiated by Patient	nursing the kidney and clearing up heatness
*232*	*D*	=Nursing the kidney, and also the skin.	Statement (elaboration)	Skin, kidney

### Communicating uncertainty to achieve consensus: Past problems and future suggestions

When a patient took an active role in directing the conversation with the TCM practitioner and act as an active participant in the communication and consultation process, he was more likely to consider his concerns to be resolved, as well as increasing his satisfaction with the TCM practitioner and the level of service he received (Finney Rutten et al., 2006; Hong and Oh, 2020). In her consultation with a patient with an issue of peeling skin, the TCM practitioner asked the patient for an update on her condition. To avoid confusion and to show her healing progress, in Table 3, lines 43 and 45, the patient invited the practitioner to view photos of the condition of her skin at the beginning of her disease. By providing visual evidence of her progress as part of her answer, the patient confirmed that the peeling issue had already improved and that they could move on to discussing the more serious problems. By documenting the improvement in her skin condition, the patient moved from an apprenticeship level to become a competent member of the care community. The patient even suggested that her condition had been improved by 80%.

**Table 3:** First example of communicating uncertainty to achieve consensus: past problems and future suggestions.

Turn	Speaker	Text	Coding	Topic
*40*	*D*	[huh (0.1) peeling: was there much peeling of the skin?	Y/N Q (exploring condition)	Skin (peeling)
*41*	*P*	Erh, the number reduced. =	Statement (comment)	Skin (peeling)
*42*	*D*	= The number reduced. =	Statement (confirmation)	Skin (peeling)
*43*	*P*	=Oh: in the beginning, do you want to take a look at my phone?=	Y/N Q (invitation to look at the skin condition)	Skin (peeling)
*44*	*D*	[I want to see	Elicited answer	Skin (peeling)
*45*	*P*	= Take a look at the time, when I took the photo, (0.1)huh:so I guess (0.2)it reduced (0.2)80%, I suppose you could say that.=	Statement (comment)	Skin (peeling)

Other than directing the conversation, when a patient provide his own recommendation, it enables them to have control over his own decisions and thus felt empowered (Burgoon et al., 1987; Finney Rutten et al., 2006; Bauer et al., 2014). In a consultation with the TCM practitioner regarding an ongoing stomach ailment, in Table 4, lines 231–233, the patient raised the possibility of a bacterial infection of ‘*Helicobacter pylori’*, as she had been intermittently experiencing the stomach issue. The TCM practitioner asked for and received the patient’s confirmation of her issue in lines 232–235. The practitioner then suggested that it was possible that she had been reinfected with Helicobacter pylori, and that her entire family should be tested for the bacteria. In this conversation, the practitioner addressed the patient’s uncertainty by listening actively and suggesting a solution. The practitioner also patiently explained a possible reason for her patient’s ongoing stomach issue and used layman’s terms to ensure a full understanding of the diagnosis. When the patients actively communicated their concerns, they were more likely to have more opportunities to resolve them. By relating her issues to the practitioner, the patient prompted her to suggest that it was likely that the patient had been reinfected by her family and that the whole family should be treated to prevent a future infection.

**Table 4:** Second example of communicating uncertainty to achieve consensus: past problems and future suggestions.

Turn	Speaker	Text	Coding	Topic
231	P	And there is another problem (.)I: I have a follow up for my stomach next month. I often easily infected with *H. pylori*.	Statement (clarification)	Stomach issue with Helicobacter pylori
232	D	Oh. Is it on and off?	Y/N Q (exploring)	Stomach issue with Helicobacter pylori
233	P	Yes. After taking many medication(.) and it will not help=	Elicited answer	Stomach issue with Helicobacter pylori
234	D	=So after the bacteria was killed, it comes again=	Statement (checking)	Stomach issue with Helicobacter pylori
235	P	=Happen again. Yes. =	Statement (confirmation)	Stomach issue with Helicobacter pylori
236	D	=Because it is also related to your family member. If you family member have *H. pylori*. (.)er.. *H. pylori*. positive, other people can infect you, I do not know whether there is still one carrier in your family, so it is useless if only you are cured, you will be infected again, that is.	Statement (comment)	Possible family members with Helicobacter pylori
237	P	Yes?	Acknowledgement	Possible family members with Helicobacter pylori.
238	D	So all of your family should do=	Statement (advice)	Possible family members with Helicobacter pylori

### Incorporating uncertainty into successful decision communication interventions during the medical consultation

When the TCM practitioner adopts a patient-centred communication style and address the patient’s uncertainty, problems are to be successfully communicated and resolved. In a consultation, patient related that she was having difficulty preparing her TCM medicine. In Table 5, lines 68 and 74, the patient repeatedly stated that she could not adequately determine the correct level of water. The TCM practitioner addressed the patient’s uncertainty and in line 73 advised her to use the width of her arm as an illustration. The practitioner further advised the patient in lines 71 and 75 to soak the medicine beforehand to avoid any issues with the level of water. By understanding the patient’s issue with the preparation of the medicine and providing alternative ways to measure the liquid, the practitioner successfully educated the patient on the correct preparation of her medicine. This type of information exchange and the provision of alternative suggestions conveyed that the practitioner was paying attention to the patient’s issues and was listening with empathy (Finney Rutten et al., 2006). The practitioner’s clear and direct explanations, such as ‘*soak it in water*’, ‘*the water level and the medicine should be the width of an arm*’ and ‘*soak it for half an hour*’ are characteristic of a patient-centred communicative mode. This type of communication painted a clear picture of what the patient needed to do to prepare her TCM medicine and had the potential to significantly lower the level of treatment avoidance (Siminoff and Fetting, 1991). It could also reduce medical mistakes made by the patients which are caused by insufficient instructions from the doctors (McLaughlin et al., 2007).

**Table 5:** Example of incorporating uncertainty into successful decision communication interventions during the medical consultation.

Turn	Speaker	Text	Coding	Topic
68	P	=So, I do not usually look at the water level. ((laugh)) So, where has the water level reached?	Elicited answer	Non-medical, preparation method of TCM med
69	D	=But usually(.) the medicine have a lot of bubbles, right?	Y/N Q (checking)	Non-medical, preparation method of TCM med
70	P	=yes yes yes		Non-medical, preparation method of TCM med
71	D	Well you (.)soak, that is to soak it in water for a while.	Statement (advice)	Non-medical, preparation method of TCM med
72	P	Yeah.		Non-medical, preparation method of TCM med
73	D	=And then, before you start boiling the medicine, you have to make sure that the water level and the medicine has a width of an arm.	Statement (advice)	Non-medical, preparation method of TCM med
74	P	Well, it teaches me one inch here. But actually, I cannot see. I have to press it ((laugh)) and then see ((laugh)) if it is one inch ((laugh)).	Statement (comment)	Non-medical, preparation method of TCM med
75	D	[It would be better if you soak it for half an hour.	Statement (advice)	Non-medical, preparation method of TCM med

## Discussion

To understand the patient-centred nature of a CoP when used in a TCM context, the analysis of participant interactions becomes significant (Van-Lier, 2000). One of the features of patient-centred care is the practitioner’s effort to draw out and corroborate the patient’s perspective to make them feel heard and understood. In TCM consultations, practitioners often use a range of linguistically strategic questions and formats to elicit the patient’s opinions, such as biomedical questions, confirmation and tracking (Zhang and Sleeboom-Faulkner, 2011; Pun, 2020). In Table 1, the practitioner reconfirmed the patient’s answer to prompt a more detailed reply to illustrate the location of the pain in the body and indicate when it is at its most extreme. By understanding the patient’s issues and perspectives, the practitioners can develop a stronger relationship with their patients by showing concern and validating their experiences. Unlike in Western Medicine, wherein the practitioners consider the scientific medical aspect, TCM practitioners would make the effort to focus on the feelings of the patients and their own experiences when giving out advice (Yip and Zhang, 2020). As a result of engaging in small talk and more personal topics, the TCM practitioner is more likely to gain a holistic understanding of the patient’s conditions and provide emotional support, thus establishing a closer relationship.

To achieve consensus, practitioners must also provide clear and understandable explanations to the patient. As the practitioner provides straightforward explanations and medical terms, patients can gradually use more TCM terminology and participate in the shared repertoire of TCM knowledge and develop their own CoP. A relationship characterised by trust and commitment can be naturally formed and fostered between TCM practitioners and patients, as the discursive practices of TCM consultations include patients in the decision-making process and provide positive emotional support and an understanding of the patient’s psychosocial context. A close relationship between patient and practitioner will result in a successful medical treatment or health outcome and increase the patient’s overall satisfaction with their care (Finney Rutten et al., 2006; Hong and Oh, 2020).

**Figure 1:**
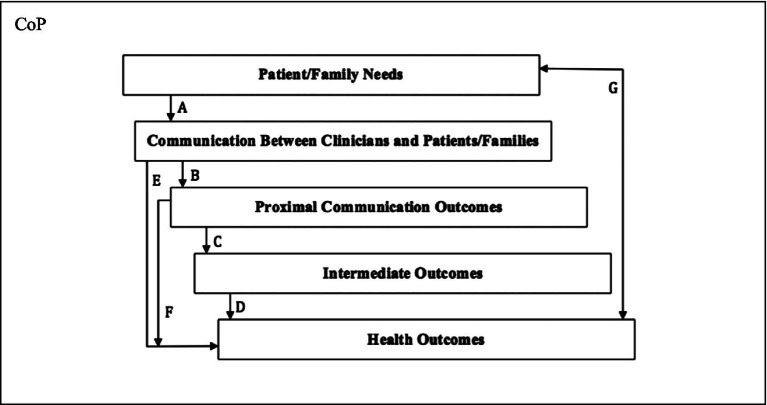
The conceptualisation of CoP in the context of patient-centred care concept [adapted from Epstein and Street (2007) as cited in Hong and Oh (2020)].

In their conceptual model explaining trust, Epstein and Street suggested that with ‘*proximal communication outcomes*’ and quality healthcare, the ‘*intermediate outcomes*’ were the major results of establishing a CoP (Li et al., 2009). Figure 1 illustrates the multiple layers of CoP outcomes derived from ‘*patient/family needs*’ and the ‘*communication between clinicians and patients/families*’. TCM practitioners typically ask their patients strategic questions to prompt them to describe their own symptoms in detail. As the length of the consultations are usually longer than those of WM practices, the TCM clinicians have more time to listen to the patient’s needs and concerns. They are subsequently able to establish a stronger relationship and a foundation for mutual trust as a part of their shared goal of improving the patient’s health. As patients and TCM physicians have a mutual understanding, a trust in one another and a strong relationship, the intermediate outcomes bridge proximal communication outcomes and health outcomes to improve the patients’ quality of life and their survival rates.

## Conclusion

This study contextualised TCM consultations and explored how patients were able to realise their CoP through their active participation and the practitioner’s patient-centeredness. This study demonstrated how the TCM practitioner used the continuous consultation method and linguistic strategies to promote communication and to gain a holistic understanding of the patient’s condition. The findings explored and illustrated the vital role played by a CoP to construct this knowledge and the patient’s participation, which assisted the TCM practitioner to better understand the patients’ conditions as a part of these meaningful conversations.

The research findings provided empirical contributions to the expanding popularity of TCM among Chinese senior citizens. By understanding how patient participation and patient-centredness develops into a CoP, the results and suggestions derived from this study will contribute to a more efficient and interactive connection between doctors and their patients. There are also several communication strategies that can be adopted by TCM doctors that can further encourage the CoP and mutual engagement. An example is asking Wh-questions which are proven to generate more elaborated answers from the patients (Pun et al., 2019a). Besides, initiating small talks can allow the practitioners to have more information about the patient’s social interaction and health condition, as well as enhancing their involvement and participation, and eventually lead to higher satisfaction of the patients (Pun et al., 2019a; Pun, 2020; Pun and Chor, 2020; Pun and Wong, 2022). We anticipate that these results will also result in further growth in the popularity of TCM.

The current research, however, is presented alongside its limitations. First, challenges of recruitment limit the scale of participants attending the project, which may further limit variation in perspectives. Also, doctor-patient communication in Chinese contexts is largely affected by power differentials between the two parties, with the doctor playing the authoritative role and the patient being silent and obedient, posing challenges to fostering patient engagement (Pun et al., 2020). The findings are consequently limited in generalizability.

Implications of this study are multi-fold. First, differences between the patient’s and practitioner’s frames for concerns during the consultation may lead to the practitioner’s inappropriate diagnosis and the patient’s noncompliance to treatment, resulting in medical failures. Thus, it is important for the practitioner to know how mutual understanding of the patient’s body conditions can be created by engagement from the two sides. Third, the holistic approach to treatment suggested in this study is important in promoting long- term relationships between practitioners and their patients. Consultation with a CoP involved can lead to better quality of interactions and reach better clinical outcomes such as high patient satisfaction and compliance with treatments.

## Data availability statement

The original contributions presented in the study are included in the article/supplementary material, further inquiries can be directed to the corresponding author.

## Ethics statement

The studies involving human participants were reviewed and approved by City University of Hong Kong. The patients/participants provided their written informed consent to participate in this study.

## Author contributions

The author confirms being the sole contributor of this work and has approved it for publication.

## Conflict of interest

The author declares that the research was conducted in the absence of any commercial or financial relationships that could be construed as a potential conflict of interest.

## Publisher’s note

All claims expressed in this article are solely those of the authors and do not necessarily represent those of their affiliated organizations, or those of the publisher, the editors and the reviewers. Any product that may be evaluated in this article, or claim that may be made by its manufacturer, is not guaranteed or endorsed by the publisher.
